# A 3-dimensional Resnet model for assessment of drug efficacy in 3D cancer models using optical coherence tomography

**DOI:** 10.1371/journal.pone.0353170

**Published:** 2026-07-24

**Authors:** Gavrielle R. Untracht, Jan Kaminski, Eike Guldenring, Boye Schnack Nielsen, Kim Holmstrøm, Katrine Hommelhoff Jensen, Peter E. Andersen

**Affiliations:** 1 Department of Health Technology, Technical University of Denmark, Kongens Lyngby, Denmark; 2 Alexandra Institute A/S, Aarhus, Denmark; 3 Bioneer A/S, Hørsholm, Denmark; Teikyo University: Teikyo Daigaku, JAPAN

## Abstract

Ninety percent of drugs fail during clinical trials, mainly due to lack of clinical efficacy. Recent developments in *in vitro* models such as 3D tumor heterospheroids have led to improvements in failure rates, but the relative lack of standardized evaluation methods for 3D cultures limits their utility in high-throughput screening. Optical coherence tomography (OCT) shows significant promise for high-throughput screening of 3D models; however, the optimal classification model and key image features for assessing drug efficacy in OCT images of spheroids has yet to be explored in detail. In this study, we investigate whether OCT combined with machine learning methods can be used to identify biomarkers of drug efficacy in 3D tumor spheroid models. We further compare the performance of two different models to determine the optimal configuration for accurate classification. Volumetric OCT images were acquired of co-cultured HT29 spheroids treated with 3 different concentrations of cisplatin. A two-dimensional multi-view ResNet model and a three-dimensional ResNet model were used to classify the images and to identify key image features associated with each group. Differences between spheroids treated with different concentrations of cisplatin are clearly visible in the OCT images. Our model was able to classify the images based on cisplatin concentration with 71.9% accuracy using the 2D multi-view model and 91.2% accuracy using the 3D model. Key features in the 3D model significantly improved the model accuracy. These results underscore the possibility that OCT could be used for high-throughput screening of drugs using 3D in vitro models and highlight key identifying features for further investigation.

## 1. Introduction

Cancer is one of the leading causes of death worldwide, accounting for approximately 10 million deaths each year [1]. While cancer deaths have declined steadily over the last few decades due to earlier diagnosis, lifestyle changes such as a reduction in smoking, and the development of novel therapies, cancer remains a significant public health challenge [2]. As our knowledge of the disease evolves, new opportunities arise for the development of targeted and personalized therapies, underscoring the need for ongoing research and development of new drugs and treatment strategies [3].

While significant investment is made towards the development of new drugs, more that 90% fail in the clinical trial stage. This puts a significant economic burden on society, leading to an average cost of between $300 million and $4.5 billion for each new drug depending on the therapeutic area, with high cost passed on to patients [4]. Further, it takes an average of 6–12 years from initial development stages until new drugs are available on the market [5]. Research has shown that any strategy to reduce the time to develop new drugs would have a significant impact both on terms of the overall cost of drugs and the number of lives saved. In particular, high-throughput screening methods that can identify the most promising candidates quickly and early in the process could have significant societal impact.

One of the main challenges affecting the large failure rate is the use of simplistic models such as 2D cell culture platforms which cannot mimic the complex tumor microenvironment found in vivo [6]. Recent advances in 3D models such as multicellular tumor spheroids have significantly improved the success rate since they can more accurately model cell-cell and cell-environment interactions [7,8]. Additionally, 3D models can incorporate scaffold structures and extracellular proteins to more accurately replicate conditions that the drugs will experience in vivo. One approach for incorporating extracellular proteins is to use heterospheroid cultures comprising co-cultures of fibroblasts and cancer cells, which simulate the tumor microenvironment more closely to the in vivo conditions compared with monocultures of cancer cells. The fibroblast cells mediate spheroid formation, and the cancer cells determine the spheroid architecture; an extracellular matrix is generated, that provides the basis for assessment of in vivo-like therapeutic efficiency of drugs [8].

Since 3D models are relatively new, few analysis methods exist that can exploit the newly available volumetric data. Bright field microscopy is commonly used to visualize spheroids, however, this technique generates 2D projection images; volume and 3D shape measurements based on bright field images are inconstant depending on the estimation method used [9]. Histological analysis is commonly employed [10,11], but the spheroids must be fixed and sliced, which can impact microstructural information. Fluorescence microscopy is a gold standard method for volumetric imaging of cells [12]. Light-sheet fluorescence microscopy, in particular, shows promise for high-throughput assessment. Although fluorescent labelling may be required to acquire images quickly with sufficient contrast, which can disturb the cellular environment, fluorescently labeled variants can readily be developed for various cell lines. However, these methods are limited in terms of imaging depth and can otherwise be challenging due to, e.g., photobleaching.

One method which shows promise towards high-throughput assessment is optical coherence tomography (OCT) [13], which enables volumetric imaging of tissue microstructures over millimeter scale volumes at video-rate imaging speeds [14]. OCT is label free, with contrast based on optical scattering, and can visualize whole spheroid volumes. Since OCT is non-destructive, it can easily facilitate longitudinal assessments.

In order to detect subtle changes in the spheroid structure, various machine learning approaches including convolutional neural networks (CNNs) have been applied to improve the detection of key features relevant for assessing drug efficacy [15–18]. Due to the complexity and computational challenges of working with large datasets, many machine learning-based classification schemes rely on single 2D images of the object [19]. However, single images may contain insufficient information for an accurate decision. This is especially true for challenging classification problems. On the other hand, full 3D datasets can be very large and computationally intensive to work with. For this reason, the 2D multi-view model was proposed. The strength of 2D multi-view CNN compared to 3D CNN is reduced computational complexity, while efficiently capturing variation in especially shape and size of the objects. Data dimensionality and required dataset size are smaller and model training time shorter. On the other hand, the 3D model can be considered the *purest* approach, where input data is a full representation of the biological object and user interference is reduced to a minimum.

In this study, we explored several ResNet [20] CNN models to quantify the structural change in HT29 heterospheroids [8] after exposure to different concentrations of cisplatin. Our goal was to develop a model that could accurately classify images of spheroids based on the amount of cisplatin and to identify key distinguishing features between the measurement classes. We here also compared the performance of the 2D multi-view model and the 3D model and report that volumetric information is a critical contribution to the accuracy of the classification task. Our overall intention is to show the potential for OCT imaging combined with deep learning as a potential tool for high-throughput screening of drug candidates without the need for human intervention.

## 2. Methods

### 2.1. Cell culture and drug treatment

Heterospheroid cultures were prepared as described in Nielsen et al. [8]. Briefly, HT-29 (ATCC®, HTB-38™) colon cancer cells were co-cultured with 1BR.3.G fibroblasts (ECACC, Cat. 90020507) in ultra-low-attachment (ULA) 96-well plates (Corning®, Corning, NY, USA, #7007). Cancer cells and fibroblasts cells were seeded in a 1:2 ratio (2500:5000) per well. Prior to intervention, the co-cultures were incubated for 72 hours to allow spheroids to form.

Dilutions of cisplatin (Merck #C2210000) were prepared from a dimethylformamide (DMF) stock solution (33.3 mM) in culture media. The vehicle suspension contained 0.3% (v/v) DMF in culture medium. At day 3 (72 hrs) after seeding each well plate was treated with the vehicle solution (20 replicate wells), the 25 µM cisplatin solution (20 replicate wells) and the 100 μM cisplatin solution (20 replicate wells), respectively.

After 4 days (96 hrs) of drug exposure the spheroids were prepared for OCT-imaging using Histogel (Thermo Scientific #HG4000012). Briefly, the growth medium (190 µl) was removed carefully from each well and 50 µl liquefied Histogel was subsequently applied to each well. Histogel was added to modulate the refractive index difference between the spheroid, the bottom of the well plate, and the background. The Histogel solidified rapidly, and the plates were transported to the OCT facility for imaging in a portable incubator chamber. A total of 180 spheroids per treatment (total number = 540 spheroids) was produced for OCT-imaging.

Validation of the effect of the drug exposure was performed by acquiring parallel bright field images of the spheroids using a NYONE^®^ Scientific Imager (Synentec GMBH, Elmshorn, Germany) and assessing the viability of the spheroids using the CellTiter-Glo^®^ 3D Cell Viability Assay (Promega #G9681) following the manufacturer’s instructions. In order to assess the significance of the change in cell viability, we have used a one-way ANOVA using Dunnett’s multiple comparison test, assuming Gaussian distribution and equal standard deviations (SD) of the data points.

### 2.2. Histology

For histological examination, 5–6 spheroids from each of the 3 treatment groups were fixed in formalin for 3 days, then dehydrated and paraffin embedded using standard procedures [8]. Five-µm-thick sections were obtained from each paraffin block and used for hematoxylin and eosin staining or immunohistochemistry. For immunohistochemical staining, sections were processed using a Ventana Discovery Ultra instrument (Roche, Basel, Switzerland). Antibodies for staining of fibroblasts and cancer cells included anti-MRC2 (mAb OTI9G4 at 1:10,000, OriGene) and anti-CK (mAb MNF116 at 1:1,000, Dako-Agilent). Both were detected with OmniMap anti-Ms HRP followed by diaminobenzidine (DAB) substrate. Sections were counterstained with hematoxylin. The stained slides were scanned using an AxioScan Z1 slide scanner, and representative images acquired using Zeiss Zen software.

### 2.3. OCT imaging

OCT images were acquired using a spectral-domain OCT system (TELESTO II, Thorlabs inc., NJ, USA). This system employs a superluminescent diode with a central wavelength of 1310 nm and a bandwidth of 140 nm and provides an imaging resolution of 5.5 μm and 13 μm in air in the axial and lateral directions, respectively. Images were acquired with a line scan rate of 28 kHz over a 1 × 1 mm area with 1 μm pixel spacing. Each well plate was imaged within one day and was kept at room temperature (21 °c) in a temperature- and humidity-controlled laboratory during imaging. One or two images were acquired of each well with different focal plane placements depending on the size of the spheroid to ensure that the classification algorithm would work independent of the focus position in the spheroid. Volumetric OCT data was pre-processed using mean-standard deviation normalization to reduce the sensitivity of the classification model to variations in image acquisition parameters.

### 2.4. Model development, testing, and validation

A 2D multi-view classification model [19] was established in python based on a ResNet architecture [21]. Sets of orthogonal, cross-sectional images were extracted from each OCT volume representing different views of the same object. The first layer of the ResNet model concatenates all features from the different views, accumulating the information coming from different angles and regions of the spheroid.

A 3D ResNet18 model was established in python by adapting the 2D model [20]. The model applies three-dimensional convolutional filters to the volumetric data samples, capturing spatial and temporal features across width, height and depth, larger and smaller structures alike.

A stratified split was used to separate data into training, validation, and test datasets. Model accuracy was calculated as the percentage of correctly classified images in the test dataset: $Accuracy=\frac{\#\;of\;true\;classifications}{total\;\#\;of\;images\;in\;the\;test\;dataset}\times \text{1}\text{0}\text{0}$. Cross-entropy loss was used to calculate the model loss: $Loss=\;-\sum_{c=\text{1}}^{m}y_{o,c}\text{ln}p_{o,c}$, where M is the number of classes (three, in this case), $y_{o,c}$ is a binary indicator of whether *c* is the correct class for observation *o*, and $p_{o,c}$ is this predicted probability that *c* is the correct class for observation *o*. Cross-entropy loss is the standard loss model for multi-class classification tasks. Confusion matrices were generated to visualize the true and false classifications stratified by class.

### 2.5. Qualitative analysis of activation maps

To gain insights into the decision-making process of the 3D CNN, we performed a qualitative analysis of super-imposed activation maps (attention maps) of the last layer of the neural network. These attention maps generated by GradCam++ [22,23] are a weighted combination of activation maps and provide insight to the most influential input regions used for the model’s prediction.

## 3. Results

### 3.1. Validation of spheroid model

**Fig 1:**
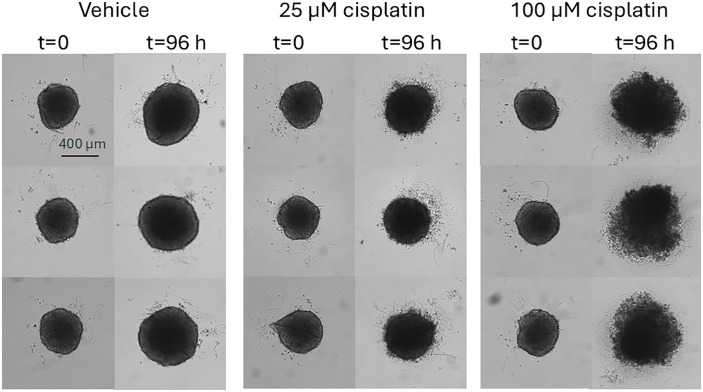
Spheroids for OCT imaging. Brightfield images of spheroids acquired after 72 h of growth before exposure to cisplatin (t = 0 h), and after 96 h of exposure to cisplatin (t = 96 h). Pairwise, images represent the same spheroid at the two time points, and for each treatment: Vehicle, 25 µM and 100 µM cisplatin, respectively. Three replicate examples are shown.

The effect of the applied cisplatin can be visualized using bright field imaging (Fig 1). Clear differences can be observed between the spheroids treated with different concentrations of cisplatin. The spheroids treated with the vehicle appear intact, but generally larger 96 h after the treatment was applied. In contrast, the spheroids treated with 25 μM cisplatin do not appear to have grown after the treatment was applied. Evidence of degradation of the spheroids is visible, including a diffuse haze and increased debris visible in the media, indicating that the spheroid structures are breaking apart. The spheroids treated with 100 μM cisplatin show further evidence of degradation, including the distorted non-spherical shape and larger hazy region of cell debris surrounding the spheroid. The images are shown in triplicate to indicate that the treatment effects are highly controlled and repeatable.

**Fig 2:**
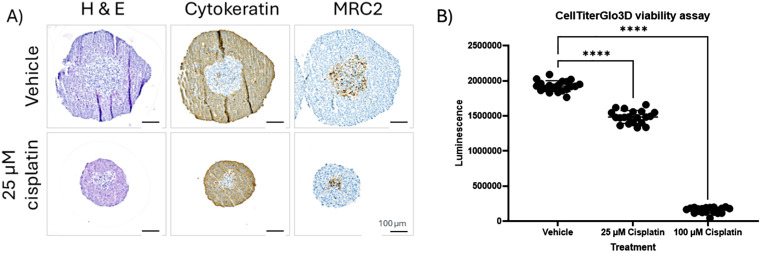
Histological assessment and viability analysis of spheroids. A) Sections of paraffin embedded spheroids were stained with hematoxylin and eosin (H&E) and using immunohistochemical (IHC) detection of cytokeratin and MRC2. Staining was achieved for detection of the cancer cells and fibroblasts, respectively. The spheroids had been treated with vehicle and 25 µM cisplatin for 96 h. B) Assessment of viability of the spheroids using the CellTiterGlo 3D Cell Viability Assay, where the ATP level is measured as an indication of metabolically active cells. For each condition (Vehicle, 25 µM and 100 µM cisplatin, respectively) 20 replicate samples were analyzed. **** indicates a statistically significant reduction in viability after cisplatin treatment at both concentrations (p < 0.0001).

The effect of cisplatin was further explored using histological analysis. The cisplatin cytotoxic agent acts by killing cells, which in the case of HT29 spheroids cause disintegration of otherwise firmly bound cells in the structures. The killed and loosened cells will be lost during the dehydration and paraffin embedding steps and are therefore poorly retained, especially at the high cisplatin dose. The spheroids treated with the vehicle are thus larger than the cisplatin-treated spheroids. Fig 2a shows the spheroids treated with the vehicle and 25 μM cisplatin stained with H&E and by immunohistochemistry for cancer cell and fibroblast markers, cytokeratin and MRC2, respectively. Histology of the spheroids treated with 100 μM cisplatin could not be performed because of the extent of degradation of the spheroids and loss of structural compactness. An attempt to embed these spheroids in paraffin after fixation was unsuccessful due to disintegration of the spheroids. Images of the vehicle-treated spheroids show that the fibroblasts assemble in the interior of the spheroid while the cancer cells form the outer shell with a few cells infiltrating the core fibroblast region. Metabolic assessment with the CellTiterGlo 3D Cell Viability Assay (Fig 2b) confirms a significant decrease in metabolic activity between all three tested groups (p < 0.0001).

**Fig 3:**
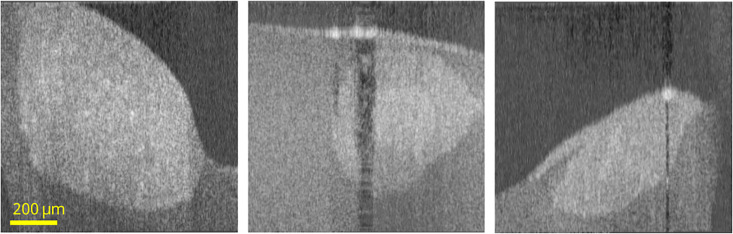
Example images of outliers that were removed from the analysis. Removed images comprised images where the spheroid was improperly placed in the well plate, for example, at the surface of the Histogel rather than embedded at the bottom of the well.

We note that, while 540 spheroids we imaged, only 467 images were included in our dataset. Images were removed from the analysis for several reasons. First, during the process of embedding the spheroids in Histogel, some of the spheroids were destroyed, leading to empty wells. Additionally, in some cases, challenges in the embedding process led to spheroids that were improperly placed in the well. These ‘outlier’ images were removed from the analysis since their inclusion significantly reduced the performance of the classifier. Some examples of images removed from the analysis are shown in Fig 3.

### 3.2. Modelling architecture

A classification model was developed using ResNet18 [21]; The ResNet is a mature CNN architecture type designed for training very deep networks effectively, readily available in PyTorch [24]. We selected ResNet18 to accommodate hardware limitations and ensure compatibility with consumer-grade systems. While deeper variants of ResNet could potentially yield improved performance, as demonstrated in other studies, our focus was on maintaining accessibility and efficiency. The CNN can efficiently capture spatial hierarchies and patterns in high-dimensional data such as image volumes and is still considered one of the leading architectures for image data classification. The imaged cancer spheroids display variation in size, shape and internal structure, both across classes and across examples from the same treatment class. As these variations do not follow a clear recognizable visual pattern, traditional feature-based methods would be less effective. For this reason, a CNN-based architecture is an ideal basis for a treatment classification model [25].

**Fig 4:**
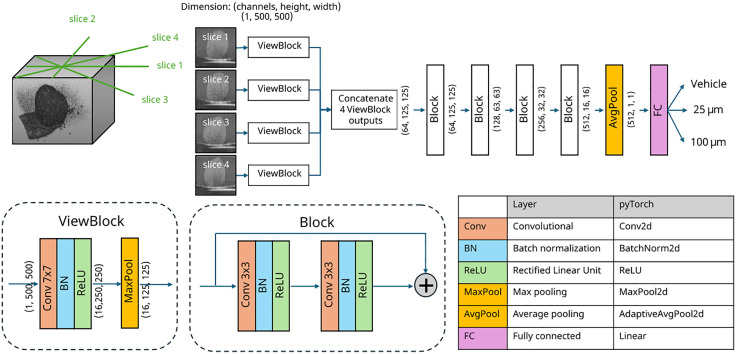
Schematic diagram of the 2D multi-view CNN model classifying the 3D OCT volumes based on drug dosages. Four, cross-sectional slices are extracted from the spheroid volume and processed by a ResNet18 model. Slices 1 and 2 are orthogonal vertical cross-sectional slices. Slices 3 and 4 are vertical cross-sectional slices atan angle of 45 degrees to slices 1 and 2. Their output feature vectors are concatenated and passed through a multi-layer perceptron to produce a final class prediction.

**Fig 5:**
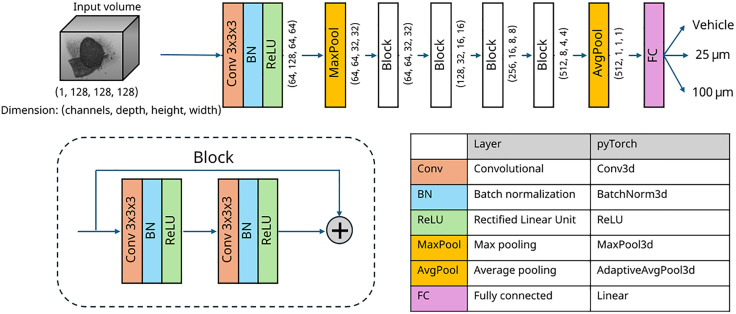
Schematic diagram of the 3D ResNet18 model classifying the 3D OCT volumes based on the drug dosages. The full 3D dataset is passed through the convolution and pooling layers of the model to extract a feature vector, which is classified by a final fully connected layer.

In order to assess the importance and influence of single voxels in the tumor spheroids on the treatment classification compared to global features such as shape and size, two different CNN designs were compared: a 2D multi-view CNN and a 3D CNN, described below. The 2D multi-view CNN processes multiple 2D slices of each sample volume, making it more light-weight computationally compared to the 3D CNN (Fig 4), both regarding memory requirements and training time. Here we can keep the original resolution and use sample slices of size 250x1000 pixels. The 3D CNN processes a resized version (described below) of the full 3D volume of the imaged cancer spheroids (Fig 5); it meets hard computational limitations related to sample volume dimensions due to the fact that multiple data samples must be loaded into GPU memory to obtain a proper batch size for the model training. On a GeForce RTX 4090 with 24GB RAM, this required a down-sampling of the training volumes to 200x500x500 voxels, representing the axial and lateral dimensions, respectively.

A stratified split was used to separate the data into training, validation and test datasets, as required for neural network model training. The splits and dataset size are described in Table 1.

**Table 1:** Data stratification for model training and validation.

	Training	Validation	Test
Vehicle	108	27	19
25 μM cisplatin	103	27	18
100 μM cisplatin	115	30	20
**Total**	**326**	**84**	**57**

### 3.3. Performance of the 2D multi-view and 3D classification models

**Fig 6:**
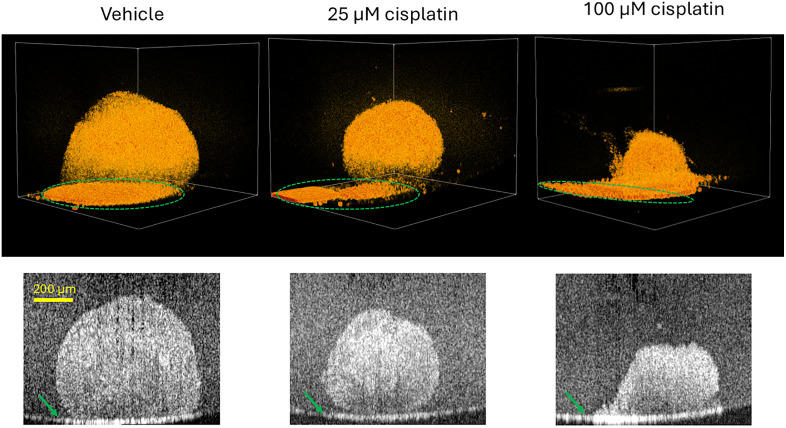
Representative class examples of OCT images of spheroids. For each condition (Vehicle, 25 µM and 100 µM cisplatin, respectively), a representative OCT image is shown Top row: 3D visualizations of the spheroid. Bottom row: 2D cross-sectional image through the center of the spheroid. The bright curved line below the spheroid images in the bottom row (green arrow) indicates the bottom of the well plate. The bottom of the well plate is also visible in the 3D visualizations, indicated by the green dotted line.

Representative examples of OCT images of the spheroids in each class are shown in Fig 6. Comparable to the brightfield images, it can be seen that the spheroid decreases in size as the concentration of cisplatin increases. Notably, the spheroids treated with 100 μM of cisplatin are the smallest since, similar to paraffin embedding, most of the killed and loosened cells were removed during the process of embedding the spheroids in Histogel.

**Fig 7:**
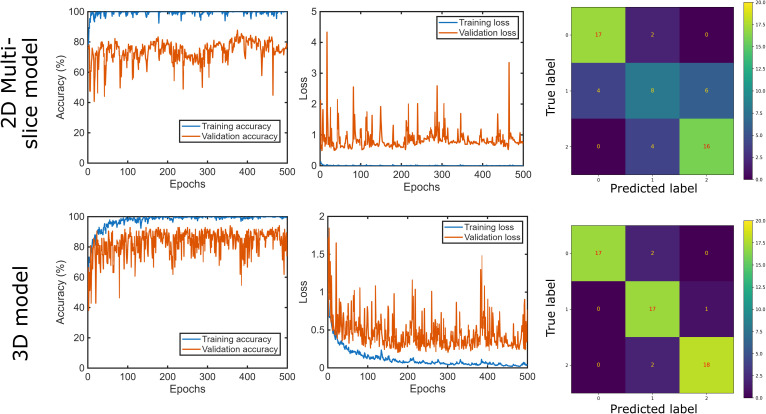
Training and validation accuracy and confusion matrices showing the performance of both classification models. Top row: 2D multi-slice model. Bottom row: 3D model.

The number of slices per volume for the best 2D multi-view model performance was experimentally found to be 4, which resulted in a model training time approximately 100 times faster than the 3D CNN. The 3D CNN model achieved an overall accuracy of 91.2% and the 2D multi-view CNN model achieved an overall accuracy of 71.9%. Learning curves and classification confusion matrices for the two models are shown in Fig 7. Although the 3D CNN dataset samples are much higher dimensional than the 2D multi-view CNN dataset samples, the training and validation loss of the 3D CNN are better decreasing towards a point of stability and show a more reasonable generalization gap between the training and validation samples.

### 3.4. Visual explanations of model predictions

**Fig 8:**
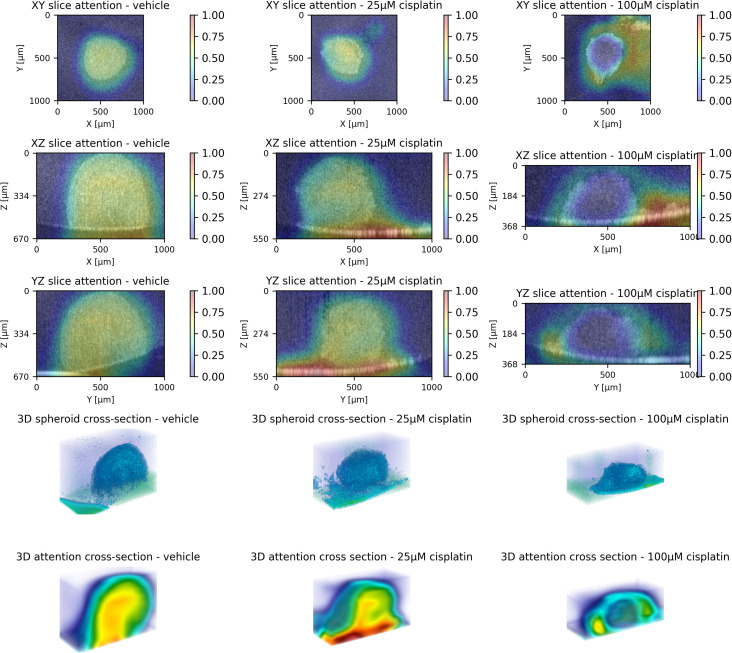
Attention maps indicating the most important spatial regions used by the model to distinguish between the classes. The top three rows show cross-sectional views of the attention maps overlayed on the spheroid images. The fourth row shows 3D images of the spheroids cut through the center for comparison with the corresponding 3D attenuation maps below. Left column Vehicle, middle column 25 µM and right column 100 µM cisplatin, respectively.

Attention maps for each spheroid were generated to confirm which features or spatial regions in the spheroid images have the largest influence on the classification task; representative 2D and 3D visualizations of the attention maps are shown for examples from each class (Fig 8). In the control group (Vehicle), the 3D model shows a clear focus on the entire spheroid, indicating that the model identifies the full structure of the spheroid as relevant. With increasing cisplatin concentrations, the focus shifts towards the debris outside of the original spheroid, indicating that the model is adapting to the presence of the drug by focusing on the degradation state of the cells. In the 25 μM case the main spheroid structure still influences the model, however, increased cell debris on the bottom of the well has emerged as a notable feature. On the other hand, for the 100 μM class, the main spheroid structure is no longer identified as a key feature in the classification task; the surrounding disintegrating cell material is a stronger identifying feature. Notably, there is no significant attention on the background, indicating that it does not influence the decision-making process.

**Fig 9:**
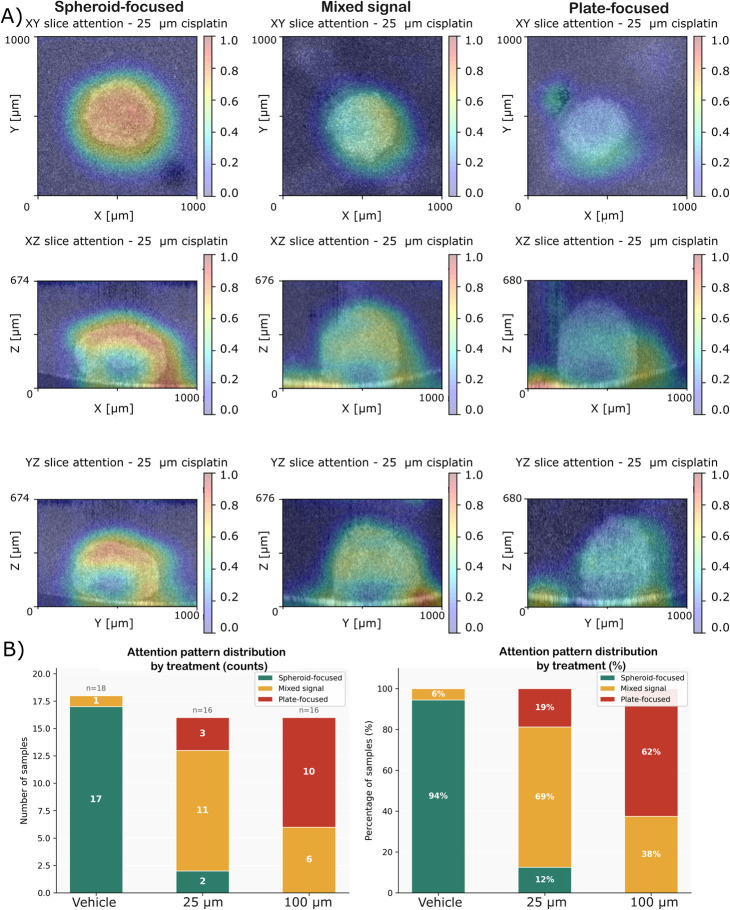
Patterns of the most important features influencing the classification task. A) Example attention maps from spheroids treated with 25 µM cisplatin indicating three recurring spatial patterns: spheroid-focused (left), mixed signal (middle), and plate-focused (right). B) Distribution of the spatial patterns stratified by treatment group.

Qualitative inspection of the attention maps across all samples reveals three recurring spatial patterns: spheroid-focused attention, plate/debris-focused attention, and a mixed distribution combining both (Fig 9). When samples from the test dataset are classified into these three categories and stratified by condition, a clear dose-dependent shift emerges. In the control group (Vehicle), spheroid-focused attention is the dominant pattern with no plate-focused cases observed. The 25 µM group introduces a substantial proportion of mixed-signal samples alongside a small number of plate-focused cases. The 100 µM group shows the highest proportion of plate-focused cases and a considerable number of mixed cases, tracking directly with the degree of morphological disintegration at increasing cisplatin dose. Taken together, this dose-dependent gradient suggests that the plate-contact region, where cell debris accumulates following cytotoxic treatment, represents a genuinely informative spatial feature rather than a simple imaging artefact, and appears to contribute to classification alongside the spheroid-focused attention signal.

## 4. Discussion

The overall aim of this study was to identify the best model for quantifying structural changes in tumor spheroids in response to an applied treatment. We tested two ResNet CNN models, a 2D multi-view model and a 3D model. The experiment shows that the ResNet CNN architecture is capable of accurately identifying different treatment-induced levels of regression in the OCT data and that both approaches have potential for serving as the basis for a complete regression prediction model. However, tradeoffs exist between the performance of the model and the required computational power. Notably, the images of the spheroids treated with 25 uM cisplatin can be classified accurately with both models even though the effect may not be visible by eye, which shows the sensitivity of our method.

### 4.1. Performance comparison between the 2D multi-view and 3D models

Comparing the two models, the biggest tradeoff is accuracy vs computational efficiency. For the 2D multi-view model, the training time was approximately 100 times faster than the 3D CNN. The 3D model required about 3 hours training time for 100 epochs on a GeForce RTX 4090. Additionally, sample size limitations required down sampling of the data. Our experiments showed that improving the pixel spacing improved the accuracy of the model, therefore we have strong evidence that running the 3D model with the full-resolution data would further improve the performance. Full resolution data could improve the accuracy or reduce the requirements for the amount of training data required. Analyzing the model further, we found that structural variation in local voxel neighborhoods of the imaged cancer spheroids are as important as global measures of size and shape, making the 3D CNN model superior for the current dataset.

We chose the ResNet architecture to allow the fairest comparison between the 2D and 3D models. In an initial phase of the study, we confirmed that neither deeper nor shallower ResNet architectures improved the performance of the 2D model (or led to less overfitted models — results not shown); in fact, use of a shallower ResNet resulted in reduced accuracy. We note that alternative architectures for the 2D model could yield better classification performance than the model implemented here and could be a topic for future investigations.

### 4.2. Model generalizability

While the training curves of both models show ability to effectively learn to fit the training data, the huge gap between the training and validation loss of the 2D multi-view CNN indicates inability to generalize well; the model is overfitting. The main factors are limited data and the complexity of the model. With more shallow architecture experiments not showing significant improvement, this could indicate that the 2D slice sets do not provide sufficient information to learn the problem efficiently, i.e., that evidence of structural patterns associated with each of the 3 classes are left out in the slice sets. For both models, the validation loss shows noisy movements compared to the training loss. This indicates an unrepresentative validation dataset that does not provide sufficient information to evaluate the ability of the model to generalize. Increasing the validation set size might help but would decrease the training set and learning abilities. Both models would benefit from larger sample datasets. Introducing data augmentation could improve the generalizability of the model without the need to acquire additional data and could be investigated to further improve the model [26].

### 4.3. Perspectives and comparison with other studies

Several studies have demonstrated the useful application of OCT to spheroid imaging including for more accurate assessment of spheroid volume [27], detection of a necrotic core region [28,29], and to investigate apoptosis and cell viability [30–32]. Functional extensions of OCT can improve the specificity towards identifying different cell types or cell viability, for example dynamic OCT has shown promise as a label-free analogue of cell viability assays [31,33,34]. Furthermore, OCT can be used to detect changes in spheroids in response to applied drugs [17,18,34–36]. Drug-induced changes can include dramatically visible effects including spheroid disintegration, or more subtle effects that cannot be easily visualized by eye. However, most of these studies used only cross-sectional B-scans in their assessments. Our study shows that volumetric data can improve classification and segmentation tasks and further refine the set of key image features and metrics relevant for assessing drug efficacy.

Overall, we see several opportunities that arise from combining OCT imaging with deep learning approaches for assessing drug efficacy. First, fully automated assessment pipelines, such as the deep learning approach presented here, are required for high-throughput screening where human assessment of images would become a speed bottleneck. Additionally, while the OCT images in our study can easily be classified by eye, not all drug effects might be as readily visible. Our study shows not only that deep learning can be used to classify images based on the drug effects, but that it can also help to identify key distinguishing features between the images. Such aspects will be critical for models that are generalized to drugs with different mechanisms of action or for classifying differences between smaller variations in drug concentrations.

### 4.4. Study strengths and limitations

Our model was deployed using consumer-grade hardware and did not rely on access to specialized computing resources such as supercomputers. This demonstrates the potential for a wider impact of the model. However, we appreciate that the model could be improved by using such specialized computing resources since they can more easily handle large datasets, leading to improved accuracy and faster training times, although such resources can be costly to access and may not be accessible to standard users.

Modeling the different levels of structural change caused by treatment can be used to estimate an appropriate treatment dose to obtain sufficient cancer regression. In our study, the models were trained on images of spheroids treated with 0, 25, and 100 μM cisplatin to identify the most accurate model. The confusion matrices show that both models, when predicting the wrong class, picks the neighbor class, i.e., an image from the control group is never falsely predicted to belong to the 100 μM cisplatin group. Also, the most accurate model, the 3D CNN, seems to be unbiased, i.e., the model is trained on all the voxels in the image rather than relying on a user-defined set of 2D slices. These factors indicate the treatment causes regression patterns, the quantity of which changes linearly with the treatment dose, which is promising for building a model of structural change for arbitrary treatment dose. However, this will require a large amount of additional image data examples involving more treatment doses. In a future study, we intend to further investigate this dose-dependent response to confirm whether the model we have developed could be used to select the optimal concentration of a drug as part of the drug screening process.

In our study, the spheroids were embedded in Histogel before imaging with OCT. This significantly improved the signal-to-noise ratio in the images, particularly by reducing the reflection from the bottom of the well plate, due to the fact that Histogel has a higher refractive index than the cell culture media. However, it also presented some challenges for the analysis. Notably, for the spheroids treated with 100 μM of cisplatin, most of the cell debris was removed during the process of embedding the spheroids in Histogel. This could indicate that some information was lost during the transfer processes and may have implications for application of the model in future experiments if Histogel is not used. Additionally, since the histogel itself scattered some light, the background noise level in the images was increased, indicating some tradeoffs with the signal improvement from using histogel.

We note that, while 540 spheroids we imaged, only 467 images were included in our dataset due to the need to remove some images where the spheroid was damaged or improporly located in the well. These ‘outlier’ images were removed from the analysis since their inclusion significantly reduced the performance of the classifier. Given the impact on the performance from the outlier removal, it is critical to have clear, objective exclusion criteria. In the future, it could be an advantage to incorporate a 2D classifier to remove outliers automatically in advance since it is a clearly visible issue with the Histogel transfer protocol.

One limitation of our method is that the model we used was trained on images of the same spheroid type that were treated with the same drug. Given the sensitivity of such classification models to small variations in images, it is unclear how easily we will be able to expand the model to recognize the response to other drugs. This step is critical to use such a model for identifying novel drug candidates. An interesting next step could be to explore how we can connect the key distinguishing features from the classifier to particular mechanisms of action of different drugs. For example, while cisplatin-induced apoptosis caused the spheroids to break apart leaving cell debris that were recognized by our model, other drugs with different mechanisms of action could have different biomarkers of efficacy that appear differently in OCT images. It would be interesting to investigate the connection between specific drug effects or mechanisms of action with their OCT image hallmarks to build a more robust and generalized model. On the other hand, this study helped us to identify the optimal hyperparameters and classification model for this type of classification tasks. Our results show that there is strong promise for the use of 3D ResNet models for drug assessment tasks in the future.

## 5. Conclusion

Our study investigated the potential of 2D and 3D ResNet models for assessing drug efficacy in 3D cell culture platforms. While both models we tested showed promise, the 3D model performed significantly better at the cost of longer training times and higher computational needs. Nonetheless, these results show the benefits of working in 3D, both in terms of the biological model and the selected imaging modality. Our results further showed that maintaining sufficient special sampling to fully resolve the OCT pattern in 3D was important to ensure the most accurate classification. In the future, we aim to expand our model to work with different cell types and drugs with different mechanisms of action. These are important next steps towards the development of a model that can be used for identifying promising new drug candidates.
